# Independent evolution of baleen whale gigantism linked to Plio-Pleistocene ocean dynamics

**DOI:** 10.1098/rspb.2017.0546

**Published:** 2017-05-24

**Authors:** Graham J. Slater, Jeremy A. Goldbogen, Nicholas D. Pyenson

**Affiliations:** 1 Department of the Geophysical Sciences, University of Chicago, Chicago, IL, USA; 2 Department of Biology, Hopkins Marine Station, Stanford University, Pacific Grove, CA, USA; 3 Department of Paleobiology, National Museum of Natural History, Washington, DC, USA

**Keywords:** macroevolution, tempo and mode, phylogeny, fossil, body size, trend

## Abstract

Vertebrates have evolved to gigantic sizes repeatedly over the past 250 Myr, reaching their extreme in today's baleen whales (Mysticeti). Hypotheses for the evolution of exceptionally large size in mysticetes range from niche partitioning to predator avoidance, but there has been no quantitative examination of body size evolutionary dynamics in this clade and it remains unclear when, why or how gigantism evolved. By fitting phylogenetic macroevolutionary models to a dataset consisting of living and extinct species, we show that mysticetes underwent a clade-wide shift in their mode of body size evolution during the Plio-Pleistocene. This transition, from Brownian motion-like dynamics to a trended random walk towards larger size, is temporally linked to the onset of seasonally intensified upwelling along coastal ecosystems. High prey densities resulting from wind-driven upwelling, rather than abundant resources alone, are the primary determinant of efficient foraging in extant mysticetes and Late Pliocene changes in ocean dynamics may have provided an ecological pathway to gigantism in multiple independent lineages.

## 1. Introduction

Vertebrates have evolved to gigantic sizes repeatedly over the past 250 Myr [1,2], reaching their extreme in today's blue whale, *Balaenoptera musculus*, which is the largest animal to have ever lived. All living baleen whales (Mysticeti), including the blue whale, are obligate suspension feeders and they possess a complex suite of adaptations that enhance the energetic efficiency of foraging on small-bodied, low trophic-level prey [3–5]. That such large animals should feed on such small prey is not without precedent; the fossil record demonstrates that large-bodied suspension feeders have arisen on several occasions in diverse clades [1,6,7]. However, the ecological mechanisms and evolutionary processes that promote and maintain gigantism remain poorly understood in general [1], and we lack a comprehensive understanding of how and when mysticetes, in particular, attained such large sizes.

Macroevolutionary analyses based on phylogenies of extant cetaceans have suggested a number of possible explanations for the evolution of mysticete gigantism, including diet-related niche partitioning among the earliest representatives of crown cetacean clades [8], rapid rates of body size evolution along the mysticete stem [9] or as one expected outcome of trade-offs between the short-term fitness benefits of size increases versus long-term costs (i.e. increased extinction risk) associated with large size [10]. While most of these hypotheses predict that gigantism should have evolved relatively early in the clade's history, as in terrestrial mammals [11], the mysticete fossil record suggests a much later origin of exceptionally large size [2,12–15] but see [16]. From their origin in the Late Eocene or Early Oligocene through the Middle Miocene, the largest mysticetes remained less than 10 m long, though several lineages appear to have independently explored the upper reaches of this size spectrum [16–18]. Lambert *et al*. [13] argued that true gigantism, defined as body lengths larger than 10 m, evolved in the early Late Miocene in response to the evolution of large macro-predatory physeteroid odontocetes and lamniform sharks. Others have suggested a later, Plio-Pleistocene origin for gigantism, either as a direct response to increased near-shore primary productivity from the Late Miocene onwards [12] or else owing to the effects of glacial cycles on habitat availability and resource distributions [14]. Despite a rich fossil record that is ideally suited for macroevolutionary inference [19], there have been no quantitative tests of when and how mysticetes achieved gigantic sizes. As such, it remains to be determined whether the evolution of blue whale-sized animals requires special explanation or whether it is simply one plausible outcome of a stochastic, constant-rates process [20].

Here, we leverage the excellent fossil record of mysticetes and a robust phylogenetic framework to provide, to our knowledge, the first formal test of when and how gigantism evolved. Using novel phylogenetic models of body size evolution and extensive simulation, we show that the evolution of exceptionally large size (more than 10 m) is a recent phenomenon that results from a fundamental clade-wide shift in the mode of body size evolution.

## 2. Material and methods

### (a) Body size data

Studies of mammalian body size evolution typically focus on body mass, given the well-defined anatomical and physiological responses of this trait to abiotic factors [11]. Reliable estimates of body mass are rare for cetaceans but available data indicate that total length (TL) scales with mass^1/3^ [21]. We therefore used log_10_(TL) as the metric for our analyses. We took a conservative approach to defining lengths for extant species by collecting length data from museum specimens, stranding records, aerial surveys and aboriginal subsistence harvests that, unlike commercial whaling records, are not subject to minimum length restrictions [22] and so do not lead to a potentially false distinction between extant and extinct body size distributions. Data collection was restricted to adult individuals, and resulted in an average *n* of 13.3 individuals per extant species (range = 1–57; electronic supplementary material, table S1).

For extinct mysticetes, we estimated log_10_(TL) in millimetres as2.1
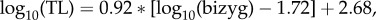
where bizyg is the bizygomatic breadth of the skull, measured in millimetres [23], which we obtained either from direct measurement of fossil specimens (by N.D.P.) or from the literature. Because this approach requires well-preserved crania, we were only able to obtain a single size estimate for each fossil species.

We computed species means for all species where *n* > 1. To account for measurement error in comparative model fitting (see below), we computed a pooled variance over all species represented by multiple specimens. The standard error of the mean for the *i*th species, including fossil taxa, was then computed as the pooled standard deviation divided by the square root of *n*_*i*_.

### (b) Phylogenetic inference

As a framework for macroevolutionary inference, we jointly estimated topology and branch lengths, in millions of years, of mysticete phylogeny from morphological and molecular data using BEAST v. 2.2.1 [24], accessed through the CIPRES Science Gateway [25]. Morphological character data for 13 extant and 63 extinct mysticetes were based on [14]. We also downloaded 11 nuclear loci and coding regions of mitochondrial genomes, where available, for all 15 extant mysticete species from Genbank (electronic supplementary material, table S1). We used the fossilized birth–death (FBD) process [26] as a prior on the distribution of branching times and branch lengths while allowing for potential ancestor-descendant relationships [27,28]. Complete details of phylogenetic analyses are provided in the electronic supplementary material.

### (c) Tempo and mode of body size evolution

We first computed two measures of phylogenetic signal (Pagel's *λ* [29] and Blomberg's *K* [30]) for log_10_(TL), using Phytools v. 0.5-38 [31] for R v. 3.3.1 [32]. We also evaluated trends in subclade disparity through time (DTT) [33] using a modified version of code from Geiger v. 2.0.6 [34]. We compared mysticete DTT to a null expectation derived from 10 000 Brownian motion (BM) simulations, and computed the morphological disparity index (MDI) as the area between the median of these simulations and the observed curve.

**Figure 1.**
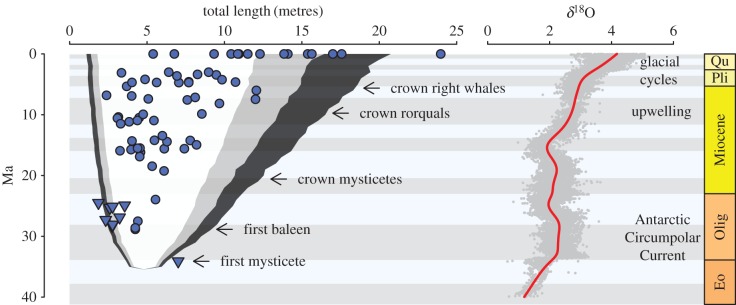
Mean body lengths for extant mysticetes and estimated length for fossil species (baleen-bearing mysticetes, circles; toothed mysticetes, triangles) are plotted according to their age as inferred from our phylogeny. Shaded areas correspond to 80 (white), 90 (grey) and 95% (black) quantiles of 1000 Brownian motion simulations on mysticete phylogeny and illustrate that the modern fauna is both lacking in small species (less than 5 m) and over-represented in large ones (more than 10 m), relative to the fossil record. To the right is a smooth-spline fitted to the Eocene–Present oxygen isotope curve [36], and used as a proxy in modelling temperature-dependent body size evolution. Higher *δ*^18^O values correspond to cooler temperatures.

To explicitly test hypotheses for the evolution of mysticete gigantism, we fitted a series of macroevolutionary models to our comparative dataset using maximum likelihood (ML). We fitted time-homogenous single rate BM, single peak Ornstein–Uhlenbeck (OU), biased random walk (also referred to as a trend model), and time-dependent rate (accelerating/decelerating: AC/DC) models of morphological evolution using the fitContinuous function in geiger. Variance of the means was added to the diagonal elements (variances) of the model-specific variance-covariance matrix during model fitting to account for measurement error. We used the OUwie.slice function in the OUwie package [35] to fit a variable rate model in which we allowed evolutionary rates to shift from one rate regime to another (either higher or lower) at some point in the past, with the shift point treated as a free parameter. We also tested for an effect of mean global ocean temperature, approximated as the δ^18^0 curve of [36], on rates of body size evolution using the fit_t_env function [37] in the RPANDA package [38]. We generated a smooth cubic spline (d.f. = 15, figure 1) from the δ^18^0 data using the smooth.spline function in the stats package in R and allowed rates of size evolution to vary as a function of this curve. Use of higher degrees of freedom, allowing for a more detailed curve, did not change ML parameter estimates or likelihoods.

We finally considered an alternative model where the mode of size evolution shifts from unbiased BM to a trended random walk at some time, t_shift_, in the past. Under this model, the elements **V**_*ij*_ of the phylogenetic variance–covariance matrix are identical to those of unbiased BM but elements in the vector of expected mean trait values **E**(*x*) are given by2.2
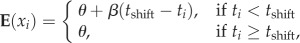
where *x*_*i*_ is the expected value of the *i*th terminal taxon, *t*_*i*_ is its occurrence time in millions of years before present, *θ* is the root state and *β* is the trend parameter, which may be positive (size increases with time) or negative (size decreases). We treated *θ*, *β*, *t*_shift_ and evolutionary rate (*σ*^2^) as free parameters in our model. Simulation tests indicate appropriate false positive rates when fitting this model, and parameter identifiability is generally good (electronic supplementary material). Relative support for each model was assessed through computation of small sample corrected Akaike weights (*w*_A_) and support for the best-fitting model over a time-homogeneous BM model was assessed via a parametric bootstrap using 1000 simulated datasets. We assessed the robustness of our results to topological and branch length variation by fitting all models to both the maximum clade credibility (MCC) tree and 1000 trees drawn at random from the post-burn in posterior sample.

### (d) Effects of preservation bias

An artificial break between the body size distributions of extant and extinct mysticetes could arise if large-bodied pelagic taxa exhibit decreased fossilization or sampling probabilities. We used a simulation approach to determine the effects of such size-biased sampling on subsequent macroevolutionary model inference when the true model of evolution is an unbiased, constant rates process. Our simulations treated sampling probability as a logistic function of size, with sampling ranging from random (P[sampling] = 0.5 for all taxa) to completely biased against large taxa (P[sampling] = 0 for any fossil taxon larger than the clade mean). Full details of our simulation procedure and methods are provided in the electronic supplementary material.

## 3. Results

### (a) Body length

Extant baleen whales have a right-shifted body size distribution compared to their fossil relatives (figure 1). Notably, this is not simply owing to the largest extant mysticetes being larger than the largest fossil species; the smallest extant mysticete, *Caperea marginata*, is larger than the smallest fossil taxa and is more comparable to the average size of mysticetes for the Oligocene through to the Pliocene.

### (b) Phylogenetic inference

**Figure 2.**
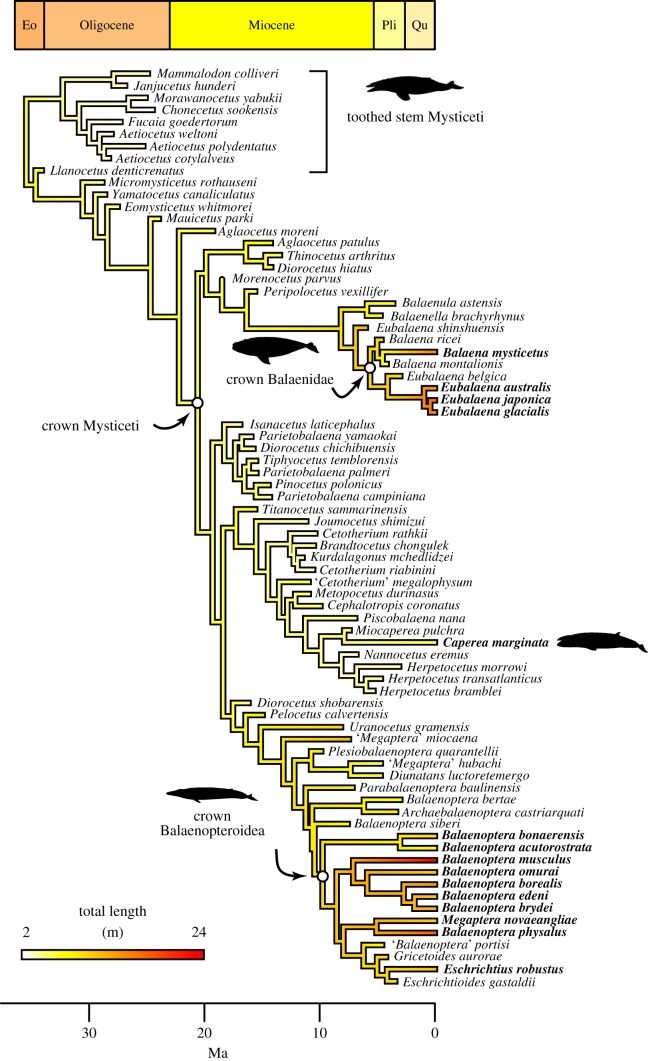
The phylogenetic distribution of large body size is young. A reconstruction of body length evolution under a simple Brownian motion model shows that large (more than 10 m) body size evolved independently in the bowhead (*Balaena*) and right (*Eubalaena*) whales, several lineages of *Balaenoptera*, and though not as pronounced, in the grey whale (*Eschrichtius*). The extant pygmy right whale *Caperea* is also large relative to its cetotheriid relatives.

The MCC tree topology is in general agreement with previous studies of both extant and fossil mysticete phylogeny [14], including the placement of *Eschrictius* and *Megaptera* within a paraphyletic *Balaenoptera* (figure 2). Divergence time estimates are somewhat younger than previous estimates derived from tip-dating [14], which probably results from use of the FBD tree prior [39]. Mapping body size onto the MCC tree topology (figure 2) confirms that increases in body size occur independently in multiple lineages of extant mysticetes.

### (c) Tempo and mode of body size evolution

**Figure 3.**
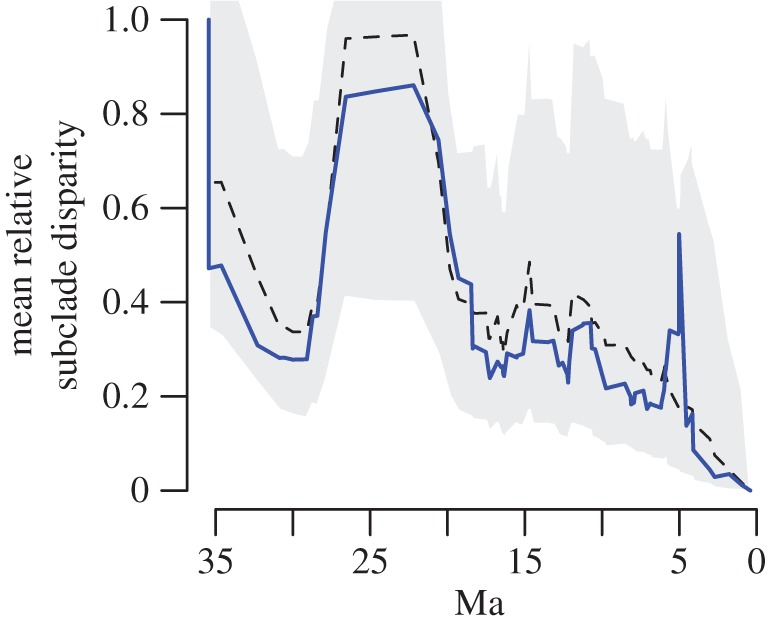
Disparity through time for mysticete body size is not entirely consistent with Brownian motion. Though the empirical curve (solid line) closely approximates the median DTT curve (dashed line) derived from Brownian motion simulation, the peak at 5.01 Ma is not consistent with such a process (MDI = 0.545, *p* = 0.064). (Online version in colour.)

Phylogenetic signal in mysticete body size is high (Pagel's *λ* = 0.94, likelihood ratio test vs *λ* = 0 *p* < 0.001; Blomberg's *K* = 0.47, *p* < 0.01). DTT analysis is mostly consistent with a constant rates process (MDI =−0.063, *p* = 0.65) but shows a pronounced pulse of increased within-clade variation at approximately 5 Ma that is inconsistent with a time-homogeneous evolutionary process (*p* = 0.066; figure 3).

**Figure 4.**
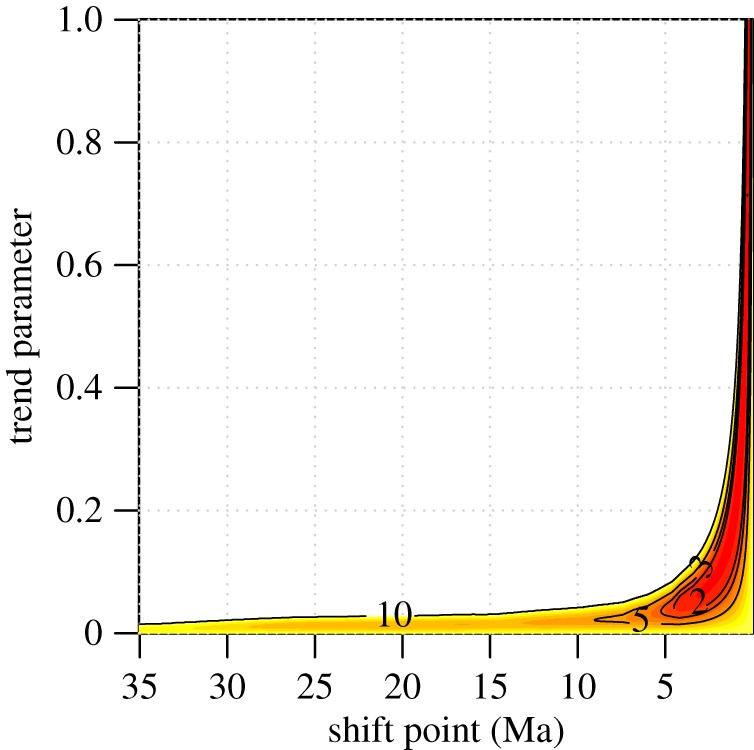
The support surface for the mode-shift model supports a young transition to gigantism. The correlation between shift-time and the trend parameter for the biased random walk phase is strong and the lack of Pleistocene fossil taxa in our phylogeny increases uncertainty in the estimation of both. Nonetheless, the 2-unit support region is sufficiently restricted to rule out traditional, pre-Pliocene explanations for gigantism. Contours show the 2, 3, 5 and 10 unit support regions.

None of the models allowing for time-dependent or temperature-dependent rates, temporal trends in the mean (e.g. Cope's rule), or a stationary optimal size provided a better fit to mysticete body size data than a simple Brownian diffusion model with a single evolutionary rate (Akaike Weight, *w*_A_, for BM = 0.33; *w*_A_ other models less than 0.26). However, we found strong support (*w*_A_ = 0.97) for the mode-shift model in which body size evolution switches from a slow unbiased random walk to an upward-biased random walk, with an inferred shift time of 0.19 Ma. Closer inspection of results revealed a saddle point in the likelihood surface for this model that is attributable to a lack of fossil data in the interval 3-0 Ma. Pleistocene glacial cycles and associated exposure and erosion of shelf area limit the preservation potential of Pleistocene cetacean fossils and little complete material is available for this interval [40]. Rather than attempting to add fragmentary Pleistocene fossil taxa to the tree, we instead reviewed the literature for fossil occurrences of extant species that could be used to truncate terminal branches and, in turn, create a Pleistocene pseudo-fossil record. Two fossil occurrences fulfilled our strict requirement of being identifiable to the species level and preserving sufficient morphology to confirm that they fall within the range of sizes exhibited by extant populations; a posterior cranium of a humpback whale (*Megaptera novaeangliae*) from the lower Kioroshi Fm Japan is dated to 0.125–0.15 Ma [41] and a grey whale (*Eschrichtius robustus*) skull and skeleton from the San Pedro Sand, California [42] is dated to 0.2–0.5 Ma [43]. Truncating the terminal branches for these two species to their youngest possible ages (0.125 and 0.2 Ma, respectively) resulted in a positive-definite Hessian matrix and increased support (*w*_A_ = 0.99; table 1) for a slightly older mode shift (0.31 Ma) with a 2-unit support range [44] of 0.13–4.5 Ma (figure 4).

**Table 1.** Parameter estimates and model fits for the truncated mysticete dataset.

model	*σ*^2^	*θ*	parameter	*t*_shift_	lnL	*k*	AICc	ΔAICc	*w*_A_
BM	0.00287	2.68	NA	—	44.39	2	−84.62	11.02	<0.01
AC/DC	0.00189	2.67	0.018	—	44.66	3	−82.98	12.65	<0.01
trend	0.00272	2.64	0.007	—	45.20	3	−84.08	13.19	<0.01
OU	0.00287	2.68	0.000	—	44.39	3	−82.45	11.56	<0.01
Temp-dep. rates	0.00072	2.68	0.002	—	44.69	3	−83.05	12.58	<0.01
rate shift	0.00234	2.67	0.003	19.92	44.54	4	−80.51	15.13	<0.01
trend shift	0.00202	2.66	0.719	0.31	52.10	4	−95.63	0.00	0.99

### (d) Effects of preservation bias

Using simulated data, we found no effect of size-biased sampling on false detection rates for the mode-shift model. This result holds even when assigning a sampling probability of zero to all fossil species larger than the clade's mean size (electronic supplementary material).

## 4. Discussion

A general difficulty, faced by both palaeontologists and biologists, in attempting to tie qualitative morphological patterns to macroevolutionary process is that multiple explanations often predict the same general outcome [45]. The mysticete fossil record qualitatively suggests a recent emergence for gigantism, and a number of compelling hypotheses have been advanced to explain this pattern, including a response to the evolution of macropredators [1,13], increases in coastal productivity [12], and glacial disruption of near-shore habitat [14]. Additional plausible explanations can be formulated that would also be consistent with the qualitative pattern but that invoke vastly different timings or processes, such as a simple increase in variance towards the present as expected under a constant rates process [46,47] or biases in the fossil record that prevent the sampling of large pelagic taxa. By taking a phylogenetic approach to modelling body size evolution in the fossil record, we have shown that none of these explanations can explain the observed discrepancy between fossil and extant mysticete body sizes and, instead, identify a shift in evolutionary mode during the past 4.5 Myr that resulted in the largest animals to have ever lived.

### (a) Time homogeneous processes and sampling bias cannot explain the late origin of gigantism

It is well known that an apparent trend towards size increase (or decrease) through time could equally be the outcome of an actual bias in the direction of size evolution or owing to an unbiased process in which variance increases through time [20,46,47]. Although mysticete body size shows high phylogenetic signal, consistent with a constant rates process [48], we found that a time homogeneous evolutionary mode is unlikely for mysticetes. Size disparity for the first 30 Myr of mysticete evolutionary history can be reasonably well approximated by simulating under BM, but the size distribution of extant species is incompatible with these expectations (figure 1). This discordance is emphasized by dramatic deviations in subclade disparity through time plots at around 5 Ma (figure 3).

We similarly find no support for the hypothesis that taphonomic biases have filtered the mysticete fossil record to an extent that generates erroneous inference of a recent mode shift. Taphonomic size biases in the terrestrial realm typically remove small species, resulting in an over-representation of large-bodied taxa [49]. Habitat preferences play an equally influential role in the marine realm [50] and could bias against the preservation of large-bodied pelagic taxa, in turn generating the disjunct body size distributions for extant and extinct mysticetes that we recover. Live–dead comparisons of extant cetacean communities using stranding records suggest that the size spectrum of fossil communities is unlikely to have been strongly filtered by taphonomic biases [15], but our simulations show that even if such filtering did occur, it cannot explain the support we recover for a shift in evolutionary mode associated with Plio-Pleistocene gigantism (electronic supplementary material). Key to understanding this initially confusing result is the nature of the discordance between fossil and extant body size distributions; while the largest extant mysticetes are larger than expected under a constant rates process, the smallest extant species are also too large (figure 1), meaning that failure to sample large fossils is only half the problem. Although a complex suite of preservation biases may have colluded to generate the patterns we observe, we are unable to conceive of such a scenario at present. Taken together, these results argue strongly against stochastic explanations for the onset of gigantism and suggest a more mechanistic explanation is required.

### (b) Timing and mechanisms

By confidently restricting the evolutionary mode shift to the Plio-Pleistocene, we are able to rule out many earlier proposed drivers of gigantism, such as the onset of an Antarctic Circumpolar Current, the initial evolution of bulk feeding or pressure from macro-predators. Because the maximum size of all described Palaeogene mysticetes is at the lower end of the Quaternary distribution [16] and well within the confidence envelope of a constant rates model, we also rule out competition among stem mysticete lineages [51] or other neocete clades [8] as a significant factor in the recent evolution of exceptional size. In fact, appending two recently described [17,18], large, stem mysticetes from the late Oligocene into our phylogeny and liberally assigning them total lengths of 10 m is insufficient to overturn strong support for a recent origin of gigantism (electronic supplementary material).

Although the Plio-Pleistocene witnessed a steep decline in average global temperatures [36], we find no support for temperature-dependent increases in macroevolutionary rate, contrary to predictions from macroecological studies [52,53] and macroevolutionary modelling of extant clades [37]. It is tempting to speculate that large size may have evolved as a directional physiological response to declining ocean temperatures rather than through increased rates that serve to increase size disparity, but the high phylogenetic signal present in our data is inconsistent with strong selection on a narrow adaptive peak [48,54]. What, then, explains the evolution of the largest animals in Earth's history?

Near-shore productivity began to increase in Late Miocene with the onset of coastal upwelling systems [55], and it seems no coincidence that mysticete palaeodiversity reached its peak during this interval [14,19,56], driven dominantly by the radiation of small-bodied and probably piscivorous [57] Cetotheriidae. Our estimated range of times for the origin of gigantism is more recent and we cannot rule out the hypothesis that disrupting effects of Pleistocene glacial cycles played a prominent role in driving the evolution of enormous size [14]. However, the world‘s oceans also experienced increasingly intensified, wind-driven upwelling dynamics from the Late Pliocene onwards [58–60]. These dynamics have been invoked as contributing to the explosive diversification of delphinine dolphins during the past 3.5 Myr [61] and are equally consistent with the timing of the shift in evolutionary mode we recover for mysticetes.

We hypothesize, based on an understanding of mysticete feeding mechanics and energenetics [5], that the origin of gigantism lies in this Late Pliocene shift in oceanography and concommitant changes in the intensity and the distribution of primary productivity. Because extant mysticetes are active bulk feeders on small-bodied prey suspended in the water column, it is high prey densities, rather than prey abundance *per se*, that increase the energetic efficiency of each feeding event [3,62]. Indeed, extended foraging bouts on highly dense and ephemeral resources are a defining characteristic of life history and feeding ecology in the largest living mysticetes, regardless of ram feeding versus lunge feeding behaviours or phylogenetic affinity [63,64]. Prior to the onset of modern upwelling regimes, the tropics appear to have been in a more or less permanent El Niño state [60], a condition that today is associated with reduced primary productivity and low densities of large mysticetes [65]. Although alkenone fluxes in sediments from the eastern equatorial Pacific suggest that productivity fluctuated with temperature during Pleistocene glacial cycles [59], the abrupt transition from homogeneous to heterogeneous, upwelling driven productivity patterns provides a direct mechanism to explain the selective advantage of large size.

The Plio-Pleistocene loss of small mysticetes (figure 1) can also be explained by these mechanisms. Our estimated timing for the origin of gigantism is coincident with a decline in global mysticete diversity from 3 Ma [14] that is driven dominantly by the near extinction of the diminutive Cetotheriidae [66], and of small, ram-feeding right whales (Balaenidae). Although intensified, wind-driven upwelling seasonally increases productivity and prey densities, the effects are localized to continental shelf breaks. Ichthyolith records from the South Pacific Gyre indicate simultaneous crashes in teleost and chondrichthyan productivity during the transition from a permanent El Niño state to modern oceanic conditions [67], further corroborating a redistribution of oceanic productivity. With the evolution of larger body sizes, mysticetes would have benefited from lower mass-specific metabolic rates to buffer against periods of low resource availability, lower costs of transport for efficient long distance migration between feeding locations, and high feeding performance when these ephemeral resources became available. At the same time, small-bodied lineages would probably have been out competed by their larger congeners that were more efficient at moving between and exploiting these patchily distributed ephemeral pulses of productivity in desert-like oligotrophic ocean ecosystems.

The lack of robustly identified Late Pliocene and Pleistocene mysticete fossils of extinct or extant species represents the final impediment to confirming our hypothesis. Deméré *et al.* [40] noted that the rich Pliocene mysticete record consists mostly of extinct but taxonomically uncertain species, but considered named fossil species from Pleistocene deposits to be *nomina dubia* that most likely represent extant taxa. Conversely, confirmed fossils of extant taxa are limited to a handful of species [41,42] and the evolutionary history of size in the largest living mysticetes remains enigmatic. Taxonomic and phylogenetic resolution of Pleistocene mysticete fossils is therefore a critical last step in confirming whether the origin of gigantism was a response to intensified upwelling and the associated restructuring of marine primary productivity during the Late Pliocene, and whether this shift in body size evolutionary dynamics occurred over a prolonged period or as a more or less instantaneous increase in size over multiple lineages.

## 5. Conclusion

By analysing the evolution of mysticete body size using phylogenetic approaches and palaeontological data, we confirm that gigantism in this clade is a surprisingly recent phenomenon. Although filter feeding using baleen had probably evolved by the Mid-Oligocene (25 Ma) [17,68], the ecological prey-scapes that energetically favour gigantism only arose in the Plio-Pleistocene, with the onset of seasonally intensified upwelling regimes (*ca* 3 Ma). As a result, we live in a time of giants; unlike any other time in geological history, modern oceans are rich with extremely large bodied suspension feeders that rely on dense but patchily distributed prey resources. Projected climate-driven changes to ocean ecosystem structure, diversity, and productivity presage a decrease in critical habitat for large-bodied baleen whales and other suspension feeding vertebrates [69], highlighting the ecological vulnerability of these giants operating on an energetic knife-edge.

## Supplementary Material

Supplementary Methods and Results: Phylogenetic Analysis, Model Performance, and Sampling Bias

## Data accessibility

Additional methods and results supporting this article have been uploaded as part of the online electronic supplementary material. All datasets and scripts used in this study are available from the Dryad Digital Repository: http://dx.doi.org/10.5061/dryad.b68g0 [70].

## References

[1] Vermeij GJ 2016 Gigantism and its implications for the history of life. PLoS ONE 11, 1–22. (10.1371/journal.pone.0146092) PMC4714876 26771527

[2] Smith FA *et al.* 2016 Body size evolution across the Geozoic. Annu. Rev. Earth Planet. Sci. 44, 523–553. (10.1146/annurev-earth-060115-012147)

[3] Goldbogen J, Calambokidis J, Oleson E, Potvin J, Pyenson N, Schorr G, Shadwick R 2011 Mechanics, hydrodynamics and energetics of blue whale lunge feeding: efficiency dependence on krill density. J. Exp. Biol. 214, 131–146. (10.1242/jeb.048157) 21147977

[4] Pyenson ND, Goldbogen JA, Vogl AW, Szathmary G, Drake RL, Shadwick RE 2012 Discovery of a sensory organ that coordinates lunge feeding in rorqual whales. Nature 485, 498–501. (10.1038/nature11135) 22622577

[5] Goldbogen J, Cade D, Calambokidis J, Friedlaender A, Potvin J, Segre P, Werth A 2017 How baleen whales feed: the biomechanics of engulfment and filtration. Annu. Rev. Mar. Sci. 9, 367–386. (10.1146/annurev-marine-122414-033905) 27620830

[6] Friedman M 2012 Parallel evolutionary trajectories underlie the origin of giant suspension-feeding whales and bony fishes. Proc. R. Soc. B 279, 944–951. (10.1098/rspb.2011.1381) PMC3259929 21849314

[7] Vinther J, Stein M, Longrich NR, Harper DAT 2014 A suspension-feeding anomalocarid from the Early Cambrian. Nature 507, 496–499. (10.1038/nature13010) 24670770

[8] Slater GJ, Price SA, Santini F, Alfaro ME 2010 Diversity versus disparity and the radiation of modern cetaceans. Proc. R. Soc. B 277, 3097–3104. (10.1098/rspb.2010.0408) PMC2982053 20484243

[9] Venditti C, Meade A, Pagel M 2011 Multiple routes to mammalian diversity. Nature 479, 393–396. (10.1038/nature10516) 22012260

[10] Clauset A 2013 How large should whales be? PLoS ONE 8, 1–6. (10.1371/journal.pone.0053967) PMC3546790 23342050

[11] Smith FA *et al.* 2010 The evolution of maximum body size of terrestrial mammals. Science 330, 1216–1219. (10.1126/science.1194830) 21109666

[12] Pyenson ND, Vermeij GJ 2016 The rise of ocean giants: maximum body size in Cenozoic marine mammals as an indicator for productivity in the Pacific and Atlantic Oceans. Biol. Lett. 12, 20160186 (10.1098/rsbl.2016.0186) 27381883 PMC4971165

[13] Lambert O, Bianucci G, Post K, de Muizon C, Salas-Gismondi R, Urbina M, Reumer J 2010 The giant bite of a new raptorial sperm whale from the Miocene epoch of Peru. Nature 466, 105–108. (10.1038/nature09067) 20596020

[14] Marx FG, Fordyce RE 2015 Baleen boom and bust: a synthesis of mysticete phylogeny, diversity and disparity. R. Soc. open sci. 2, 140434 (10.1098/rsos.140434) 26064636 PMC4448876

[15] Pyenson ND 2011 The high fidelity of the cetacean stranding record: insights into measuring diversity by integrating taphonomy and macroecology. Proc. R. Soc. B 278, 3608–3616. (10.1098/rspb.2011.0441) PMC3189373 21525057

[16] Tsai C-H, Kohno N 2016 Multiple origins of gigantism in stem baleen whales. Sci. Nat. 103, 89 (10.1007/s00114-016-1417-5) 27717969

[17] Tsai C-H, Fordyce RE 2015 The earliest gulp-feeding mysticete (Cetacea: Mysticeti) from the Oligocene of New Zealand. J. Mamm. Evol. 22, 535–560. (10.1007/s10914-015-9290-0)

[18] Tsai C, Fordyce R 2016 Archaic baleen whale from the Kokoamu Greensand: earbones distinguish a new late Oligocene mysticete (cetacea: Mysticeti) from New Zealand. J. R. Soc. New Zealand 46, 117–138. (10.1080/03036758.2016.1156552)

[19] Uhen MD, Pyenson ND 2007 Diversity estimates, biases, and historiographic effects: resolving cetacean diversity in the Tertiary. Palaeontol. Electron. 10, 11A–22.

[20] Raup DM, Gould SJ 1974 Stochastic simulation and evolution of morphology-towards a nomothetic paleontology. Syst. Zool. 23, 305–322. (10.2307/2412538)

[21] Lockyer C 1976 Body weights of some species of large whales. J. Conseil 36, 259–273. (10.1093/icesjms/36.3.259)

[22] Gambell R 1993 International management of whales and whaling: an historical review of the regulation of commercial and aboriginal subsistence whaling. Arctic 46, 97–107. (10.14430/arctic1330)

[23] Pyenson ND, Sponberg SN 2011 Reconstructing body size in extinct crown Cetacea (Neoceti) using allometry, phylogenetic methods and tests from the fossil record. J. Mamm. Evol. 18, 269–288. (10.1007/s10914-011-9170-1)

[24] Bouckaert R, Heled J, Kühnert D, Vaughan T, Wu C-H, Xie D, Suchard MA, Rambaut A, Drummond AJ 2014 BEAST 2: a software platform for Bayesian evolutionary analysis. PLoS Comput. Biol. 10, e1003537 (10.1371/journal.pcbi.1003537) 24722319 PMC3985171

[25] Miller MA, Pfeiffer W, Schwartz T 2010 Creating the CIPRES Science Gateway for inference of large phylogenetic trees. In Gateway Computing Environments Workshop (GCE), 2010, New Orleans, LA, USA, pp. 1–8.

[26] Heath TA, Huelsenbeck JP, Stadler T 2014 The fossilized birth–death process for coherent calibration of divergence-time estimates. Proc. Natl Acad. Sci. USA 111, E2957–E2966. (10.1073/pnas.1319091111) 25009181 PMC4115571

[27] Gavryushkina A, Welch D, Stadler T, Drummond AJ 2014 Bayesian inference of sampled ancestor trees for epidemiology and fossil calibration. PLoS Comput. Biol. 10, e1003919 (10.1371/journal.pcbi.1003919) 25474353 PMC4263412

[28] Gavryushkina A, Heath TA, Ksepka DT, Stadler T, Welch D, Drummond AJ 2016 Bayesian total-evidence dating reveals the recent crown radiation of penguins. Syst. Biol. 66, 57–73. (10.1093/sysbio/syw060) PMC5410945 28173531

[29] Pagel M 1997 Inferring evolutionary processes from phylogenies. Zool. Scr. 26, 331–348. (10.1111/j.1463-6409.1997.tb00423.x)

[30] Blomberg SP, Garland T, Ives AR 2003 Testing for phylogenetic signal in comparative data: behavioral traits are more labile. Evolution 57, 717–745. (10.1111/j.0014-3820.2003.tb00285.x) 12778543

[31] Revell LJ 2012 phytools: an R package for phylogenetic comparative biology (and other things). Methods Ecol. Evol. 3, 217–223. (10.1111/j.2041-210X.2011.00169.x)

[32] R Core Team. 2016 R: a language and environment for statistical computing. Vienna, Austria: R Foundation for Statistical Computing.

[33] Harmon LJ, Schulte JA, Larson A, Losos JB 2003 Tempo and mode of evolutionary radiation in iguanian lizards. Science 301, 961–964. (10.1126/science.1084786) 12920297

[34] Pennell MW, Eastman JM, Slater GJ, Brown JW, Uyeda JC, FitzJohn RG, Alfaro ME, Harmon LJ 2014 geiger v2.0: an expanded suite of methods for fitting macroevolutionary models to phylogenetic trees. Bioinformatics 30, 2216–2218. (10.1093/bioinformatics/btu181) 24728855

[35] Beaulieu JM, Jhwueng D-C, Boettiger C, O'Meara BC 2012 Modeling stabilizing selection: expanding the Ornstein-Uhlenbeck model of adaptive evolution. Evolution 66, 2369–2383. (10.1111/j.1558-5646.2012.01619.x) 22834738

[36] Zachos JC, Dickens GR, Zeebe RE 2008 An early Cenozoic perspective on greenhouse warming and carbon-cycle dynamics. Nature 451, 279–283. (10.1038/nature06588) 18202643

[37] Clavel J, Morlon H 2017 Accelerated body size evolution during cold climatic periods in the Cenozoic. Proc. Natl Acad. Sci. USA 114, 4183–4188. (10.1073/pnas.1606868114) 28373536 PMC5402425

[38] Morlon H, Lewitus E, Condamine FL, Manceau M, Clavel J, Drury J 2016 RPANDA: an R package for macroevolutionary analyses on phylogenetic trees. Methods Ecol. Evol. 7, 589–597. (10.1111/2041-210X.12526)

[39] Matzke NJ, Wright A 2016 Inferring node dates from tip dates in fossil Canidae: the importance of tree priors. Biol. Lett. 12, 20160328 (10.1098/rsbl.2016.0328) 27512133 PMC5014026

[40] Deméré TA, Berta A, McGowen MR 2005 The taxonomic and evolutionary history of fossil and modern balaenopteroid mysticetes. J. Mamm. Evol. 12, 99–143. (10.1007/s10914-005-6944-3)

[41] Nagasawa K, Mitani Y 2004 A humpback whale, *Megaptera novaeangliae* (Borowski, 1781), from the Pleistocene Kioroshi Formation of Inba-mura, Chiba Prefecture, central Japan. Paleontol. Res. 8, 155–165. (10.2517/prpsj.8.155)

[42] Barnes LG, McLeod S 1984 The fossil record and phyletic relationships of gray whales. In The gray whale *Eschrictius robustus* (eds ML Jones, SL Swartz, S Leatherwood), pp. 3–32. San Diego, CA: Academic Press.

[43] Pyenson ND, Lindberg DR 2011 What happened to gray whales during the Pleistocene? the ecological impact of sea-level change on benthic feeding areas in the North Pacific Ocean. PLoS ONE 6, 1–14. (10.1371/journal.pone.0021295) PMC3130736 21754984

[44] Edwards AW 1992 Likelihood, expanded edn Baltimore, MD: Johns Hopkins University Press.

[45] Foote M 1996 Models of morphological diversification. In Evolutionary paleobiology (eds D Jablonski, DH Erwin, JH Lipps), pp. 62–88 Chicago, IL: University of Chicago Press.

[46] Gould SJ 1988 Trends as changes in variance: a new slant on progress and directionality in evolution. J. Paleontol. 62, 319–329. (10.1017/S0022336000059126)

[47] Jablonski D 1997 Body-size evolution in Cretaceous molluscs and the status of Cope's rule. Nature 385, 250–252. (10.1038/385250a0)

[48] Revell LJ, Harmon LJ, Collar DC 2008 Phylogenetic signal, evolutionary process, and rate. Syst. Biol. 57, 591–601. (10.1080/10635150802302427) 18709597

[49] Behrensmeyer AK, Western D, Boaz DED 1979 New perspectives in vertebrate paleoecology from a Recent bone assemblage. Paleobiology 5, 12–21. (10.1017/S0094837300006254)

[50] Kidwell SM, Holland SM 2002 The quality of the fossil record: implications for evolutionary analyses. Annu. Rev. Ecol. Syst. 33, 561–588. (10.1146/annurev.ecolsys.33.030602.152151)

[51] Tsai C-H, Ando T 2016 Niche partitioning in Oligocene toothed mysticetes (Mysticeti: Aetiocetidae). J. Mamm. Evol. 23, 33–41. (10.1007/s10914-015-9292-y)

[52] Botero C, Dor R, McCain C, Safran RJ 2014 Environmental harshness is positively correlated with intraspecific divergence in mammals and birds. Mol. Ecol. 23, 259–268. (10.1111/mec.12572) 24283535

[53] Lawson AM, Weir J 2014 Latitudinal gradients in climatic-niche evolution accelerate trait evolution at high latitudes. Ecol. Lett. 17, 1427–1436. (10.1111/ele.12346) 25168260

[54] Hansen TF 1997 Stabilizing selection and the comparative analysis of adaptation. Evolution 51, 1341–1351. (10.2307/2411186) 28568616

[55] Jacobs DK, Haney TA, Louie KD 2004 Genes, diversity, and geologic process on the Pacific coast. Annu. Rev. Earth Planet. Sci. 32, 601–652. (10.1146/annurev.earth.32.092203.122436)

[56] Quental TB, Marshall CR 2010 Diversity dynamics: molecular phylogenies need the fossil record. Trends Ecol. Evol. 25, 434–441. (10.1016/j.tree.2010.05.002) 20646780

[57] Collareta A *et al.* 2015 Piscivory in a Miocene Cetotheriidae of Peru: first record of fossilized stomach content for an extinct baleen-bearing whale. Sci. Nat. 102, 1–12. (10.1007/s00114-015-1319-y) 26553062

[58] Marlow JR, Lange CB, Wefer G, Rosell-Melé A 2000 Upwelling intensification as part of the Pliocene-Pleistocene climate transition. Science 290, 2288–2291. (10.1126/science.290.5500.2288) 11125138

[59] Lawrence KT, Liu Z, Herbert TD 2006 Evolution of the eastern tropical Pacific through Plio-Pleistocene glaciation. Science 312, 79–83. (10.1126/science.1120395) 16601186

[60] Fedorov AV, Dekens PS, McCarthy M, Ravelo AC, deMenocal PB, Barreiro M, Pacanowski RC, Philander SG 2006 The Pliocene Paradox (mechanisms for a permanent El Niño). Science 312, 1485–1489. (10.1126/science.1122666) 16763140

[61] do Amaral KB, Amaral AR, Ewan Fordyce R, Moreno IB In press. Historical biogeography of delphininae dolphins and related taxa (artiodactyla: Delphinidae). J. Mamm. Evol. (10.1007/s10914-016-9376-3)

[62] Hazen EL, Friedlaender AS, Goldbogen JA 2015 Blue whales (*Balaenoptera musculus*) optimize foraging efficiency by balancing oxygen use and energy gain as a function of prey density. Sci. Adv. 1, e1500469 (10.1126/sciadv.1500469) 26601290 PMC4646804

[63] Wishner KF, Schoenherr JR, Beardsley R, Chen C 1995 Abundance, distribution and population structure of the copepod *Calanus finmarchicus* in a springtime right whale feeding area in the southwestern Gulf of Maine. Cont. Shelf Res. 15, 475–507. (10.1016/0278-4343(94)00057-T)

[64] Croll DA, Marinovic B, Benson S, Chavez FP, Black N, Ternullo R, Tershy BR 2005 From wind to whales: trophic links in a coastal upwelling system. Mar. Ecol. Progress Ser. 289, 117–130. (10.3354/meps289117)

[65] Benson SR, Croll DA, Marinovic BB, Chavez FP, Harvey JT 2002 Changes in the cetacean assemblage of a coastal upwelling ecosystem during El Niño 1997–98 and La Niña 1999. Progress Oceanogr. 54, 279–291. (10.1016/S0079-6611(02)00054-X)

[66] Boessenecker RW 2013 Pleistocene survival of an archaic dwarf baleen whale (Mysticeti: Cetotheriidae). Naturwissenschaften 100, 365–371. (10.1007/s00114-013-1037-2) 23525578

[67] Sibert E, Norris R, Cuevas J, Graves L 2016 Eighty-five million years of Pacific Ocean gyre ecosystem structure: long-term stability marked by punctuated change. Proc. R. Soc. B 283, 20160189 (10.1098/rspb.2016.0189) PMC4892790 27194702

[68] Peredo CM, Pyenson ND, Boersma AT 2017 Decoupling tooth loss from the evolution of baleen in whales. Front. Mar. Sci. 4, 67 (10.3389/fmars.2017.00067)

[69] Hazen EL *et al.* 2013 Predicted habitat shifts of Pacific top predators in a changing climate. Nat. Clim. Change 3, 234–238. (10.1038/nclimate1686)

[70] Slater GJ, Goldbogen JA, Pyenson ND 2017 Data from: Independent evolution of baleen whale gigantism linked to Plio-Pleistocene ocean dynamics. *Dryad Digital Repository*. (10.5061/dryad.b68g0) PMC5454272 28539520

