# Supplementary material for: Independent evolution of baleen whale gigantism linked to Plio-Pleistocene ocean dynamics
Source: Proc Biol Sci. 2017 May 24;284(1855):20170546. doi: 10.1098/rspb.2017.0546 (PMC5454272; doi:10.1098/rspb.2017.0546)
Supplement: Supplementary Methods and Results: Phylogenetic Analysis, Model Performance, and Sampling Bias [file rspb20170546supp1.pdf]

Supplementary phylogenetic methods and simulation results for Independent evolution of whale gigantism linked with Plio-Pleistocene ocean dynamics

Graham J. Slater[1], Jeremy A. Goldbogen[2], Nicholas D. Pyenson[3]

[1]Department of the Geophysical Sciences, University of Chicago, Chicago IL, USA

[2]Department of Biology, Hopkins Marine Station, Stanford University, Pacific Grove CA, U.S.A.

[3]Department of Paleobiology, National Museum of Natural History, Washington DC. USA.

Phylogenetic Inference

Morphological character data for living and fossil mysticetes were sourced from Marx and Fordyce's[1] comprehensive treatment, with the following edits and additions. All undescribed or unidentified fossil taxa (n=13) were removed, as were the 3 odontocete taxa. We retained the archaeocete *Zygorhiza kochii*. We also replaced the composite taxon *Eubalaena* spp. with coded characters for the three extant *Eubalaena* species. These edits resulted in 3 invariant characters (characters 63 - 65) that were previously used to resolve odontocete relationships, and which were subsequently deleted from the character matrix. The final morphological matrix contained 76 taxa (13 extant, 63 extinct) coded for 269 characters.

We downloaded 11 nuclear loci (*AMBN* exons 6 and 13, *ATP7A*, *BDNF*, *CSN2*, *DMP1*, *ENAM*, *PRM1*, *RAG1*, *SRY*, and *TBX4*) and complete mitochondrial genomes, where available, for all 15 extant mysticete species from Genbank (Table S1). Sequences were aligned using `MUSCLE`[2] through `Geneious` v. 8.0[3] and checked by eye. We extracted only protein coding genes from mitochondrial genomes, ensuring that reading frames were maintained and that overlapping regions were assinged to one gene only. We then used `PartitionFinder` v 1.1.1[4] to determine the optimal partitioning scheme for the 11 nuclear loci and 3 mitochondrial partitions, corresponding to 1st through 3rd codon positions, under the Bayesian Information Criterion.

We simultaneously inferred phylogeny and branch lengths for living and extinct mysticetes using `BEAST` 2.2.1[5], accessed through the CIPRES Science Gateway[6]. Five morphological partitions, each corresponding

to characters with the same number of states, were assigned Markov models with an adjustment for ascertainment bias due to sampling of variable characters only[7]. Molecular data were partitioned and assigned models of molecular evolution based on `PartitionFinder` results. We used the fossilized birth death process[8] as a prior on the tree topology and allowed for sampled ancestor-descendant relationships[9,10]. Fossil species were assigned uniform age ranges, following Marx and Fordyce[1]. We placed an exponential prior with mean = 1 on net diversification rate, a Uniform(0, 1) prior on turnover rate, and $\beta(2, 2)$ on sampling proportion. For the relaxed molecular clock, we placed a lognormal prior on the mean of lognormal distribution from which branch rates were sampled ($\mu = -3.5, \sigma = 1$) and a gamma distribution on the standard deviation ($\alpha = 0.5396, \beta = 0.3819$). For the relaxed morphological clock, we applied a lognormal prior ($\mu = -2.12, \sigma = 1$) on the mean of the distribution, and the same shape gamma prior as for the molecular clock on the standard deviation. Preliminary analyses indicated slow mixing of tree topology, resulting in prohibitively long runs that failed to converge and recovered unusual relationships among living and extant species. To ameliorate this effect, we provided a user-specified starting tree based on a single maximum likelihood search performed in `raxML` v 7.4.2[11] without partitioning of the data. This was sufficient to improve topological and parameter mixing in our BEAST analyses. We ran four chains of 100 million generations, sampling every 10,000 steps. Convergence was assessed using `Tracer` v1.6 and the four chains subsequently combined to generate effective sample sizes of >200 for all parameters. We finally computed a Maximum Clade Credibility (MCC) tree from the combined output using `TreeAnnotator`.

Testing the identifiability of the mode-shift model

We examined reliability of parameter estimates under the mode-shift model by simulating trait evolution on our mysticete phylogeny using randomly sampled values of t_{shift} and β, and then fitting the model to these data. For t_{shift}, the relationship between estimated and true values is significant ($r^2 = 0.87, p < 0.001$), and has a slope of 0.93, but the intercept is shifted upwards to 1.26, indicating a slight bias towards inferring ages that are too old for more recent shifts. This is somewhat corrected when weighting by model fit ($r^2 = 0.95, a = 0.61, b = 0.97$). Plotting true versus estimated parameter values (Fig S1a) reveals that the deviation is strongest for shifts occurring in the interval 0-2.5 Ma, with MLEs diverging both upward and downward. This localized effect, which is related to the lack of fossil data in this interval (see main text), can be confirmed as repeating the regression with shifts younger than 2.5Ma removed moves the intercept closer to zero (unweighted regression: $a = 0.33$, $b = 0.97$; weighted regression: $a = 0.12$, $b = 0.99$). Estimated shift times remain within the 0– 2.5Ma interval in almost all cases where the true shift also lies in that interval, indicating that an inferred shift within this time frame can be conservatively interpreted as real, despite

a lack of precision in identifying the exact timing. A least-squares regression of estimated values of β on true values is also significant ($r^2 = 0.52, p < 0.001$) and gives both intercept and slope close to expected values of [0,1] (a= 0.02, b=0.96) indicating that this parameter is reliably estimated in most cases (Fig S1b). Weighting the regression by Akaike weight of the mode shift model (i.e., down weighting the influence of parameter estimates from poor fits) increases the overall fit of the regression ($r^2 = 0.67, a = 0.013, b = 0.99$).

Size Biased Sampling

To test for an effect of size-baised sampling, specifically biases against sampling large bodied taxa, we took a simulation approach. We simulated phylogenies under a constant rates birth-death process ($\lambda = 0.2$, $\mu = 0.15$) for 35 time units, the approximate age the mysticete stem, using the `pbtree` function of the `phytools` package and retained only those trees that contained more than 100 tips of which exactly 15 were extant, to yield fair comparisons to our empirical data-set. We then forward simulated Brownian motion using the `fastBM` function in `phytools` at a rate of 0.003. For each simulated tree we generated 6 "sampled" datasets, in which extant taxa are always sampled, but the probability of a fossil species being sampled was a logistic function of size. Specifically,

$$P(\text{sampling}) = 1 - \frac{1}{1 + e^{c(0.5-x)}} \ , \qquad (1)$$

where c is the scale of the logistic function (i.e. the steepness of the curve about the midpoint), and x is size of the fossil species after transforming all (living and extinct) species' sizes on the range [0,1]. The size-dependent probability of sampling is determined by the steepness of the curve. We considered $c \in \{0, 2.5, 5, 10, 25, 100\}$ (fig S2). When $c = 0$, $P(\text{sampling}) = 0.5$ for all fossil taxa, regardless of size, but increasing values of c result in increased sampling probabilities for small taxa and decreased sampling probabilities for larger taxa. When $c = 100$, $P(\text{sampling}) \approx 0$ for all taxa larger than the midpoint of the size range (Fig. S4). We fitted BM and mode-shift models to each of the 6 sampled datasets, plus the completely sampled dataset and computed Akaike weights, w_A, for each model for each comparison. We assessed how sampling affects model selection by regressing $w_A$for the mode-shift model on sampling scheme for each simulated dataset and examining the distribution of slopes; if biased sampling leads to improved fit for the mode-shift model over the true BM model, we would expect this distribution of slopes to trend positive. We also computed the standardized effect size of sampling bias on model selection, treating the completely sampled data set as the control group. Finally, we computed false positive rates, measured as the proportion of simulations for which the trend shift model received higher support, for $w_A = \{0.5, 0.75, 0.9, 0.99\}$ over all levels of sampling bias.

A t-test suggests a slight but significant bias towards positive slopes for regressions of w_A on sampling bias ($\bar{b} = 0.0063, t = 4.36, p < 0.001$; figure S3a). However, standardized effect sizes of sampling bias on model selection, treating the completely sampled data set as the control group, indicate that all levels of sampling except the most severe tend to increase support for the true BM model, rather than the mode-shift model (figure S3b). Furthermore, the effect size for a strict bias against sampling large taxa ($c = 100$) is so small as to be essentially negligible ($d = 0.041$).

Effects of Unsampled Fossil Taxa

Our use of Marx and Fordyce's[1] character matrix allowed us to maximize taxonomic sampling but could bias us towards inferring a recent shift in evolutionary mode if large Paleogene taxa were not sampled. Two recently described fossils from the late Oligocene of New Zealand present such a conundrum. *Horopeta umarere*[12] and *Whakakai waipata*[13] are both relatively large taxa (estimated total lengths of around 6.5m) but the holotype specimens present incompletely fused cranial sutures, suggesting sub-adult individuals that presumably attained larger lengths at completely maturity. Unfortunately, these taxa were coded in their original publications for a different character matrix[14], preventing simple integration into our analyses.

To determine whether these taxa could overturn the inference of a Plio-Pleistocene shift to gigantism, we took the simple but liberal approach of repeating analyses after appending these two taxa to our maximum clade credibility tree and assigning them a total length of 10m. We assumed that these two fossil taxa are sister taxa and placed them, in turn as the sister clade to a clade consisting of *Mauicetus*, *Aglaocetus* and crown mysticetes, but one node crownward than *Eomysticetus*. This placement is consistent with the most parsimonious solutions reported in Tsai and Fordyce[13] and ensured that both terminals fell within the stratigraphic ranges of the fossil taxa (27–25 Ma). By liberally assigning both taxa a total length of 10m, we account for the possibility that very large mysticetes were present by the late Oligocene and that the pathway to gigantism was set at this time[15]. Instead, we find no real effect of including these taxa: support for the mode shift model declines marginally from $w_A = 0.99$ to $w_A = 0.97$ but the maximum likelihood estimate for the shift time in this most supported model remains at 0.3Ma. While future fossil discoveries may tip the balance towards an earlier origin of modern body sizes, the current mysticete record is inconsistent with a Paleogene onset for gigantism.

References

[1] Marx, F. G. & Fordyce, R. E., 2015 Baleen boom and bust: a synthesis of mysticete phylogeny, diversity and disparity. *Royal Society open science* **2**, 140434.

[2] Edgar, R. C., 2004 MUSCLE: multiple sequence alignment with high accuracy and high throughput. *Nucleic acids research* **32**, 1792–1797.

[3] Kearse, M., Moir, R., Wilson, A., Stones-Havas, S., Cheung, M., Sturrock, S., Buxton, S., Cooper, A., Markowitz, S., Duran, C. *et al.*, 2012 Geneious Basic: an integrated and extendable desktop software platform for the organization and analysis of sequence data. *Bioinformatics* **28**, 1647–1649.

[4] Lanfear, R., Calcott, B., Ho, S. Y. & Guindon, S., 2012 PartitionFinder: combined selection of partitioning schemes and substitution models for phylogenetic analyses. *Molecular biology and evolution* **29**, 1695–1701.

[5] Bouckaert, R., Heled, J., Kühnert, D., Vaughan, T., Wu, C.-H., Xie, D., Suchard, M. A., Rambaut, A. & Drummond, A. J., 2014 BEAST 2: a software platform for Bayesian evolutionary analysis. *PLoS Comput Biol* **10**, e1003537.

[6] Miller, M. A., Pfeiffer, W. & Schwartz, T., 2010 Creating the CIPRES science gateway for inference of large phylogenetic trees. In *Gateway Computing Environments Workshop (GCE), 2010*, pp. 1–8. IEEE.

[7] Lewis, P. O., 2001 A likelihood approach to estimating phylogeny from discrete morphological character data. *Systematic Biology* **50**, 913–925. (doi:10.1080/106351501753462876).

[8] Heath, T. A., Huelsenbeck, J. P. & Stadler, T., 2014 The fossilized birth–death process for coherent calibration of divergence-time estimates. *Proceedings of the National Academy of Sciences* **111**, E2957–E2966.

[9] Gavryushkina, A., Welch, D., Stadler, T. & Drummond, A. J., 2014 Bayesian inference of sampled ancestor trees for epidemiology and fossil calibration. *PLoS Comput Biol* **10**, e1003919.

[10] Gavryushkina, A., Heath, T. A., Ksepka, D. T., Stadler, T., Welch, D. & Drummond, A. J., 2016 Bayesian total-evidence dating reveals the recent crown radiation of penguins. *Systematic Biology* (doi: 10.1093/sysbio/syw060).

[11] Stamatakis, A., 2006 Raxml-vi-hpc: maximum likelihood-based phylogenetic analyses with thousands of taxa and mixed models. *Bioinformatics* **22**, 2688–2690.

[12] Tsai, C.-H. & Fordyce, R. E., 2015 The earliest gulp-feeding mysticete (Cetacea: Mysticeti) from the Oligocene of New Zealand. *Journal of Mammalian Evolution* **22**, 535–560. ISSN 1573-7055. (doi: 10.1007/s10914-015-9290-0).

[13] Tsai, C. & Fordyce, R., 2016 Archaic baleen whale from the kokoamu greensand: earbones distinguish a new late oligocene mysticete (cetacea: Mysticeti) from new zealand. *Journal of the Royal Society of New Zealand* **46**, 117–138.

[14] Marx, F. G., 2011 The more the merrier? a large cladistic analysis of mysticetes, and comments on the transition from teeth to baleen. *Journal of Mammalian Evolution* **18**, 77–100.

[15] Tsai, C.-H. & Ando, T., 2016 Niche partitioning in Oligocene toothed mysticetes (Mysticeti: Aetiocetidae). *Journal of Mammalian Evolution* **23**, 33–41. ISSN 1573-7055. (doi:10.1007/s10914-015-9292-y).

Table S1: Total length data for living and fossil mysticetes and GenBank accession data for molecular sequences. Total length is converted to meters for ease of reading.

species	log_{10} TL(cm)	StdErr	TL (m)	n	amb exon 6	amb exon 13	ATP7A	BDNF	CSN2	Enam	PRM1	RAG1	SRY	TBX-4	mtGenome
Aetiocetus cotylalveus	2.44	0.068	2.75	1											
Aetiocetus polydentatus	2.41	0.068	2.6	1											
Aetiocetus weltoni	2.44	0.068	2.77	1											
Aglaocetus moreni	2.79	0.068	6.1	1											
Aglaocetus patulus	2.89	0.068	7.79	1											
Archaebalaenoptera castriarquati	2.92	0.068	8.26	1											
Balaena montalionis	2.69	0.068	4.87	1											
Balaena mysticetus	3.19	0.010	15.38	50	EU444974	EU444998	EU444963	EU444888	EU444900	EU445060	EU444938	EU445024	AB108509	-	NC_005268
Balaena ricei	3.03	0.068	10.71	1											
Balaenella brachyrhynus	2.85	0.068	7.05	1											
Balaenoptera acutorostrata	2.83	0.023	6.76	7	EU444971	EU444995	EU444960	EU444885	EU444897	EU445057	EU444935	EU445021	AB108510	AB279634	AP006468
Balaenoptera bertae	2.81	0.068	6.4	1											
Balaenoptera bonaerensis	2.97	0.030	9.33	5	EU444970	EU444994	EU444959	EU444884	EU444896	EU445056	EU444934	EU445020	AB275391	AB279635	NC_006926
Balaenoptera borealis	3.15	0.048	14.09	2	EU444968	EU444992	EU444957	EU444882	EU444894	EU445054	EU444932	EU445018	-	AB279636	NC_006929
Balaenoptera brydei	3.09	0.018	12.3	15	-	-	-	-	-	-	-	-	AB275392	AB279637	NC_006928
Balaenoptera edeni	3.04	0.030	10.94	4	EU444969	EU444993	EU444958	EU444883	EU444895	EU445055	EU444933	EU445019	AB275393	-	NC_007938
Balaenoptera musculus	3.38	0.030	23.97	5	EU444967	EU444991	EU444956	EU444881	EU444893	EU445053	EU444931	EU445017	AB108511	AB279638	NC_001601
Balaenoptera omurai	3.02	0.024	10.43	8	-	-	-	-	-	-	-	-	-	AB279639	NC_007937
Balaenoptera physalus	3.24	0.030	17.57	5	EU444966	EU444990	EU444955	EU444880	EU444892	EU445052	EU444930	EU445016	AB108512	AB279641	NC_001321
"Balaenoptera" portisi	2.89	0.068	7.7	1											
Balaenoptera siberi	2.88	0.068	7.6	1											
Balaenula astensis	2.84	0.068	6.93	1											
Brandtocetus chongulek	2.65	0.068	4.49	1											
Caperea marginata	2.73	0.020	5.39	12	EU444973	EU444997	EU444962	EU444887	EU444899	EU445059	EU444937	EU445023	-	-	NC_005269
Cephalotropis coronatus	2.68	0.068	4.75	1											
"Cetotherium" megalophysum	2.74	0.068	5.45	1											
Cetotherium rathkii	2.50	0.068	3.15	1											
Cetotherium riabinini	2.49	0.068	3.09	1											
Fucaia goedertorum	2.37	0.068	2.33	1											
Chonecetus sookensis	2.27	0.068	1.86	1											
Diorocetus chichibuensis	2.60	0.068	3.97	1											
Diorocetus hiatus	2.74	0.068	5.48	1											
Diorocetus shobarensis	2.66	0.068	4.58	1											
Diunatans luctoretemergo	2.89	0.068	7.7	1											
Eomysticetus whitmorei	2.64	0.068	4.41	1											
Eschrichtioides gastaldii	2.98	0.068	9.47	1											
Eschrichtius robustus	3.06	0.017	11.51	15	EU444972	EU444996	EU444961	EU444886	EU444898	EU445058	EU444936	EU445022	-	AB279643	NC_005270
Eubalaena australis	3.14	0.009	13.85	57	EU444975	EU444999	EU444964	EU444889	EU444901	EU445061	EU444939	EU445025	AB108514	AB279631	NC_006930
Eubalaena belgica	2.95	0.068	8.98	1											
Eubalaena glacialis	3.19	0.034	15.64	3			-	-	-	GQ354840	GQ368527	GQ368546	-	-	X75587*
Eubalaena japonica	3.23	0.068	17	1	EU444976	EU4445000	EU444964	EU444889	EU444901	EU445062	EU444939	EU445025	AB275390	AB279632	NC_006931
Eubalaena shinshuensis	3.08	0.068	12.06	1											
Gricetoides aurorae	2.99	0.068	9.84	1											
Herpetocetus bramblei	2.57	0.068	3.69	1											
Herpetocetus morrowi	2.52	0.068	3.35	1											
Herpetocetus transatlanticus	2.61	0.068	4.03	1											
Isanacetus laticephalus	2.66	0.068	4.53	1											
Janjucetus hunderi	2.51	0.068	3.23	1											
Joumocetus shimizui	2.59	0.068	3.85	1											
Kurdalagonus mchedlidzei	2.52	0.068	3.3	1											
Llanocetus denticrenatus	2.85	0.068	7.01	1											
Mammalodon colliveri	2.55	0.068	3.56	1											
Mauicetus parki	2.74	0.068	5.55	1											
"Megaptera" hubachi	2.75	0.068	5.62	1											
"Megaptera" miocaena	3.08	0.068	12	1											
Megaptera novaeangliae	3.04	0.030	10.86	5	EU444965	EU444989	EU444954	EU444879	EU444891	EU445051	EU444929	EU445015	AB108513	AB279642	AP006467
Metopocetus durinasus	2.64	0.068	4.38	1											
Micromysticetus rothauseni	2.63	0.068	4.24	1											
Miocaperea pulchra	2.71	0.068	5.08	1											
Morawanocetus yabukii	2.44	0.068	2.77	1											
Morenocetus parvus	2.73	0.068	5.33	1											
Nannocetus eremus	2.38	0.068	2.38	1											
Parabalaenoptera baulinensis	2.91	0.068	8.11	1											
Parietobalaena campiniana	2.61	0.068	4.03	1											
Parietobalaena palmeri	2.66	0.068	4.54	1											
Parietobalaena yamaokai	2.51	0.068	3.25	1											
Pelocetus calvertensis	2.91	0.068	8.2	1											
Peripolocetus vexillifer	2.79	0.068	6.12	1											
Pinocetus polonicus	2.80	0.068	6.29	1											
Piscobalaena nana	2.60	0.068	4.01	1											
Plesiobalaenoptera quarantellii	2.93	0.068	8.54	1											
Thinocetus arthritus	2.78	0.068	5.99	1											
Tiphyocetus temblorensis	2.64	0.068	4.39	1											
Titanocetus sammarinensis	2.87	0.068	7.41	1											
Uranocetus gramensis	2.99	0.068	9.68	1											
Yamatocetus canaliculatus	2.63	0.068	4.24	1											

Table S2: False positive rates for the mode shift model under different sampling biases and for different Akaike weight (w_A) cut-offs.

w_A	complete	0	2.5	5	10	25	100
0.5	0.253	0.177	0.183	0.192	0.225	0.259	0.286
0.75	0.094	0.062	0.069	0.072	0.093	0.136	0.154
0.9	0.033	0.031	0.026	0.038	0.047	0.064	0.076
0.95	0.017	0.019	0.016	0.017	0.022	0.039	0.046
0.99	0.005	0.004	0.006	0.003	0.009	0.01	0.016

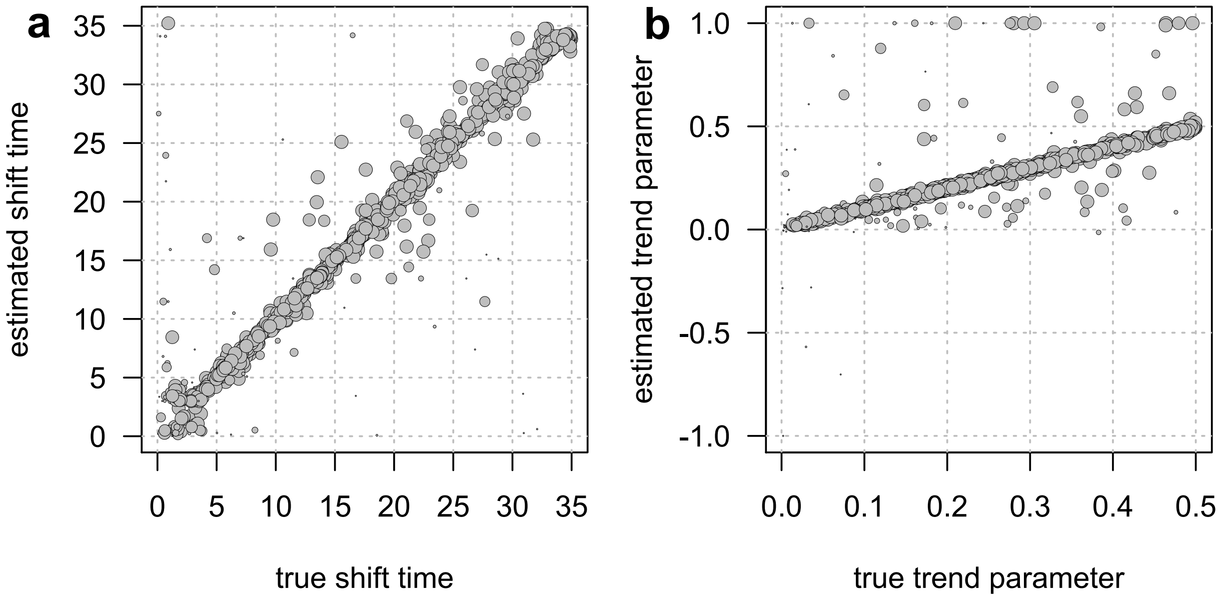

Figure S1: **Parameters of the mode-shift model can be reliably estimated on the mysticete phylogeny**. **a.** Estimated shift times show a 1:1 correspondence with true shift times over most of the phylogeny. Shifts younger than 2.5Ma are more difficult to estimate accurately, but most estimates fall within the 0–2.5Ma interval. **b.** Estimates of the trend parameter show less deviation. Symbol size corresponds to Akaike Weight (w_A for the mode-shift model.

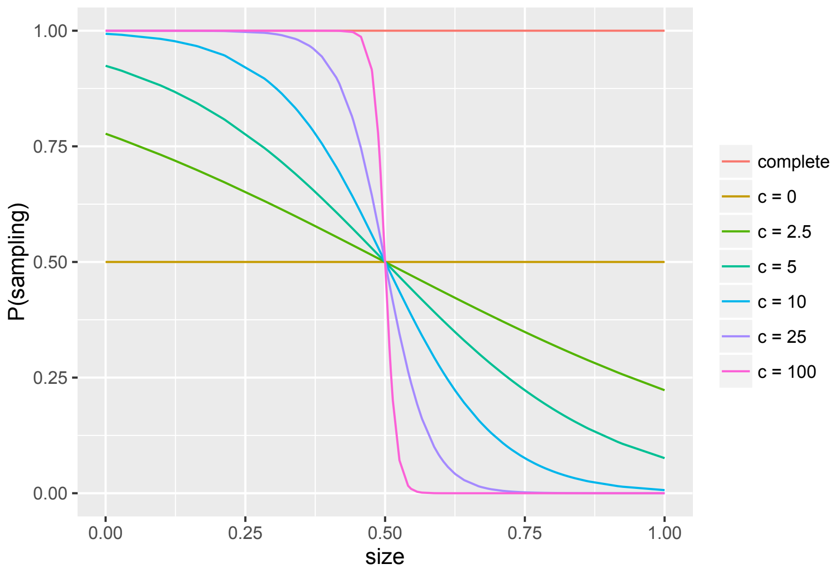

Figure S2: Logistic model of sampling for body size. Complete sampling recovers all taxa, regardless of size, with probability = 1, while a logistic slope of zero samples all taxa, regardless of size with probability = 0.5. Slopes > 0 result in increasing size biases. Note that the value of the logistic function $f(x)$ increases with size and thus $P(\text{sampling}|x) = 1 - f(x)$.

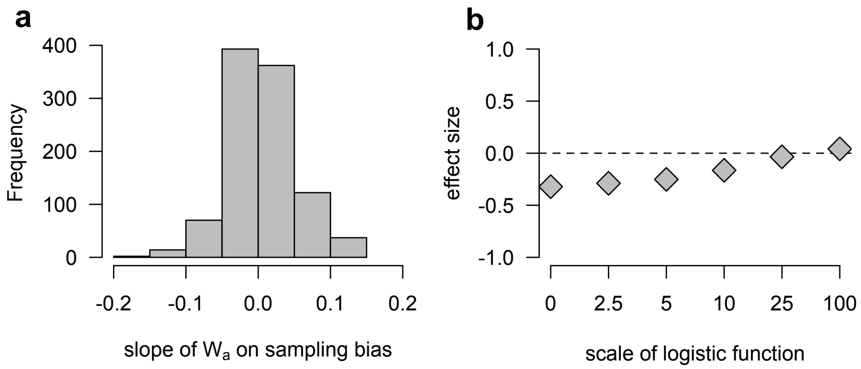

Figure S3: Sampling biases cannot explain preference of the mode-shift model over a simple Brownian motion model. a) Although there is a tendency to increase support for the mode relative to BM as sampling becomes more biased, this result appears to be driven by the fact that random but incomplete sampling of the fossil record increases relative support for BM over a mode shift. b) Standardized effect sizes (Cohen's d) relative to complete sampling show that this effect is driven by a return of support to complete sampling levels, and that there is no strong bias effect towards the mode shift model.
